# Interest of 3D Fat-Saturated T1-Weighted Magnetic Resonance Imaging for the Assessment of Cervical Artery Dissection

**DOI:** 10.5334/jbr-btr.867

**Published:** 2016-02-11

**Authors:** Yasser Bencheikh, Pola Kulczycka, Francesco Macri, Domitille Millon, Joel Greffier, Jean Paul Beregi, Ahmed Larbi

**Affiliations:** 1 CHU Nîmes, FR; 2 Cliniques Universitaires Saint Luc, BE

**Keywords:** Brain, cerebral ischemic stroke, vascular dissection, MRI

A 43–years–old woman without any medical history or particular treatment consulted the emergency room for fluctuant headache, developing for five days and resistant to usual treatment. The headache was associated with hypoesthesia of the left part of the face with ptosis. Clinical examination found a slight hypoesthesia in V2 and V3 territories and a Bernard-Horner’s syndrome. Brain magnetic resonance imaging (MRI) showed no brain parenchymal abnormality, especially on diffusion-weighted imaging. Cervical vessels imaging with axial fat suppressed spin echo T1-weighted sequence showed a pre occlusive dissection of petrous segment of the left carotid artery. Study of the cervical portion was suboptimal due to the magnetic susceptibility artifact of a dental implant (Figure 1A).

**Figure 1A:**
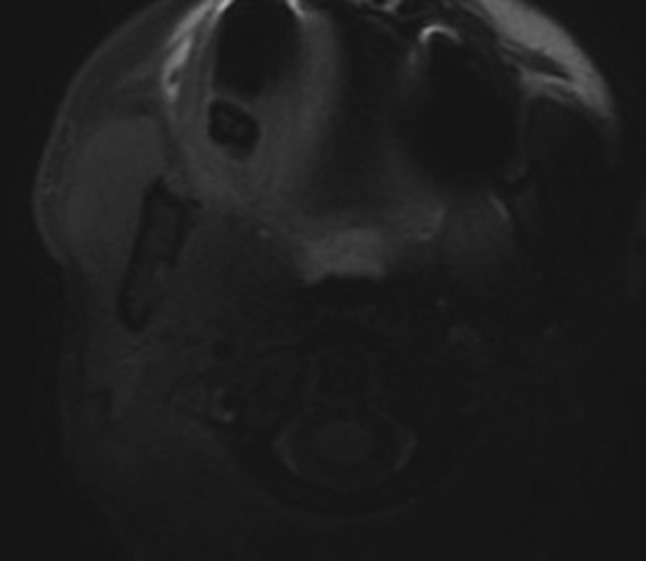
Cervical MRI, axial fat suppressed SE T1-weighted sequence. Studying the cervical portion of left carotid artery is hampered by magnetic susceptibility artifact due to the presence of dental implant.

A complementary 3D volumetric fat suppressed spin echo T1-weighted sequence was realized and allowed to partially overcome this artifact and to bring out the extension of this dissection to the cervical part of the carotid artery (Figure 1B, arrow). MR angiography with injection of gadolinium chelates confirmed a tapered stenosis of the left carotid artery (Figure 1C, arrows). The patient was treated with efficient anticoagulant therapy.

**Figure 1B:**
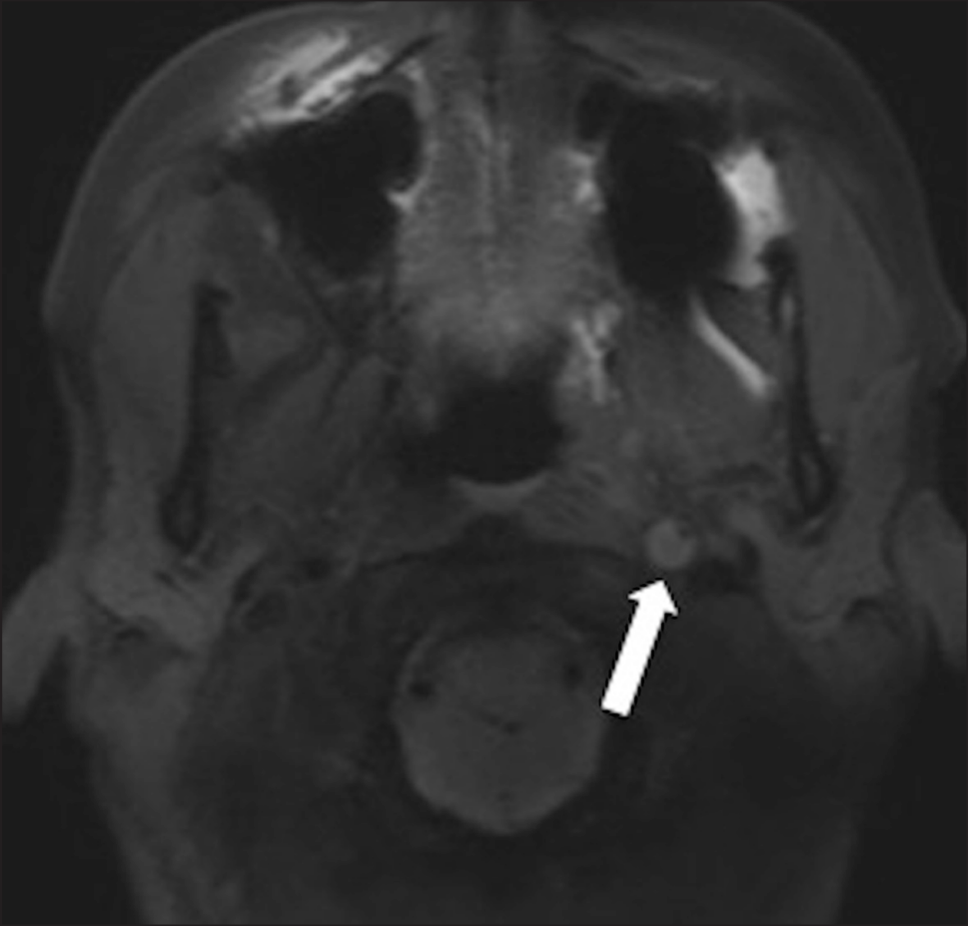
Cervical MRI, volumetric 3D fat suppressed SE T1-weighted sequence allowing to overcome the magnetic susceptibility artifact and to bring out extension of the cervical portion of left internal carotid artery dissection (arrow).

**Figure 1C:**
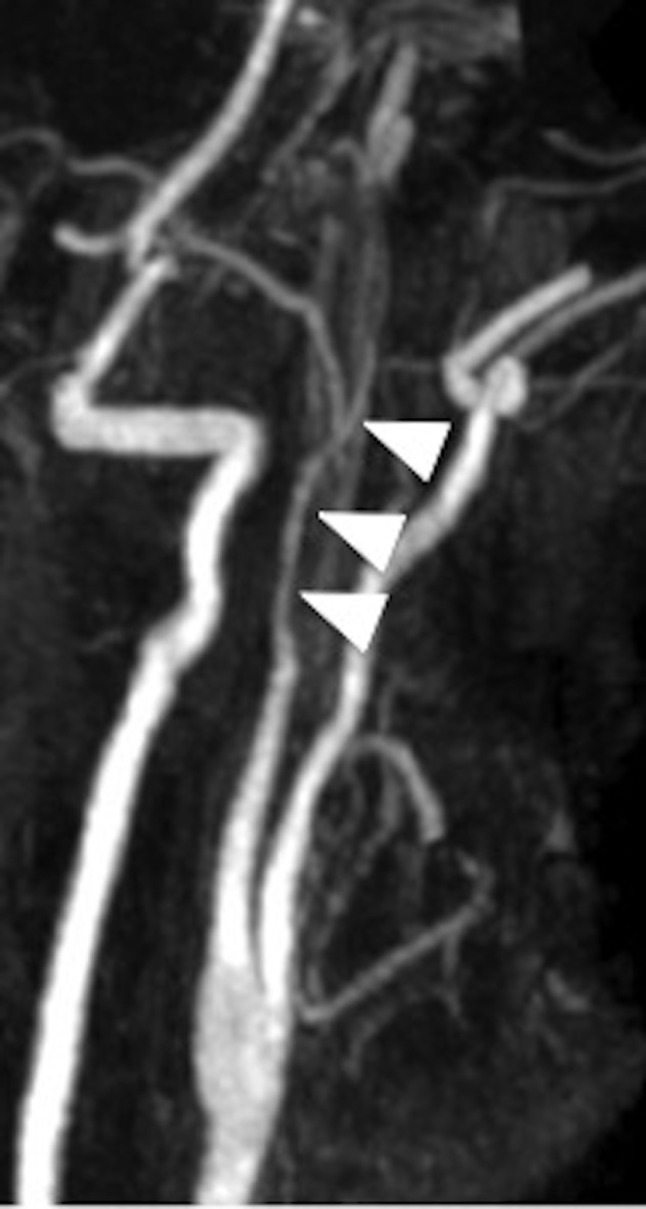
Gadolinium chelates enhanced MR angiography confirming the stenosis of the tapered left internal carotid artery (arrowheads).

## Comment

Cervical arteries dissections are due to development of hematoma in media layer of the vessel wall. They represent the principal cause of cerebral ischemic strokes in young adults (20%), but the prognosis remains good. They predominate in the carotid arteries, especially in the petrous segment and can be multiple in a third of cases.

The role of imaging is fundamental for the diagnosis and the follow-up of both the dissection and the related cerebral ischemic lesions. Indeed, MRI allows at a time analysis of the wall and of the lumen.

The axial fat suppressed SE T1-weighted sequence (Figure A) represents the sequence of choice, showing T1-weighted, crescent-shaped, high signal intensity in the vessel wall (corresponding to subacute hematoma) with endoluminal narrowing. However, this sequence has many limits—as in the case of vessel sinuosity—especially near to bony structures or foreign bodies (Figure B). It also presents poor quality paravertebral venous flow suppression. Recent studies have reported some advantages of the 3D volumetric fat suppressed SE T1-weighted sequence [1]. This sequence allows optimal coverage of all arterial portions (cervical and intracranial) in only one acquisition (Figure C) and also saves precious time in the case of brain ischemic stroke and decreasing artifacts due to patient movements. Moreover, this sequence enables realizing multiplanar reformatting, which allows a better view on extension of the wall-hematoma, especially when studying tortuous vessels. It is known that fat suppression quality clearly decreases in lower cervical level. This volumetric sequence provides satisfying fat signal suppression in perivascular thereby allowing fine analysis of the V1 segment in vertebral arteries. Lastly, it seems less prone to magnetic susceptibility artifacts caused by metallic material as illustrated by our case.

## Competing Interests

The authors declare that they have no competing interests.
